# Schizophrenia and myasthenia gravis: a case report

**DOI:** 10.1192/j.eurpsy.2023.2229

**Published:** 2023-07-19

**Authors:** D. Bošnjak Kuharić, T. Sabo, A. Jambrošić Sakoman, M. Herceg

**Affiliations:** 1 Department for psychotic disorders- female; 2 Deparment for addiction treatment, University Psychiatric Hospital Vrapce, Zagreb, Croatia

## Abstract

**Introduction:**

Despite a variety of pharmacological and psychotherapeutic interventions, treatment of schizophrenia can still be challenging, even more when certain comorbidities are present. Myasthenia gravis (MG) is an autoimmune disorder of the neuromuscular junctions caused by antibodies against acetylcholine and tyrosine kinase. While co-occurrence of schizophrenia and MG is rare, treatment can be complicated as specific treatment of one condition can lead to worsening of other (e.g. anticholinergic side effects of psychopharmacotherapy, psychiatric side effects of corticosteroids).

**Objectives:**

To discuss treatment difficulties in the case of a patient with schizophrenia and multiple somatic comorbidities, including MG.

**Methods:**

A case report and a review of literature.

**Results:**

We report a case of a 50-year-old female patient who was admitted to psychiatric hospital due to psychotic decompensation presented with dysphoria, paranoid delusions, agitation, verbal aggression and hostility. Clinical presentation and psychopharmacological treatment were complicated with her comorbid disorders, MG, which was recently treated because of a relapse, and hypothyroidism, which worsened as she neglected her regular check-ups. Multidisciplinary approach was needed to control the symptoms of her comorbid disorders, which, especially MG, limited psychopharmacological options. Combination of antipsychotics (aripiprazole, haloperidol) and mood stabilizer (sodium valproate) led to clinical improvement of psychotic symptoms. However, poor insight remained- the patient insisted on demission and was not interested in suggested psychotherapeutic and sociotherapeutic programs.

**Conclusions:**

In complex cases like this, multidisciplinary approach is essential for adequate treatment of both psychiatric and comorbid somatic disorders. Conditions like MS can prolong treatment or even worsen the symptoms of a psychiatric disorder, especially since they limit the use of psychopharmacotherapy. Due to this, psychotherapeutic interventions could be even more important to keep a stable remission with a good insight and adherence to both psychiatric and somatic treatment.

**Disclosure of Interest:**

None Declared

